# Compensatory UTE/T2W Imaging of Inflammatory Vascular Wall in Hyperlipidemic Rabbits

**DOI:** 10.1371/journal.pone.0124572

**Published:** 2015-05-15

**Authors:** Bongjune Kim, Jaemoon Yang, Young Han Lee, Myeong-Hoon Kim, Dan Heo, Eugene Lee, Jin-Suck Suh, Seungjoo Haam, Yong-Min Huh

**Affiliations:** 1 Department of Chemical and Biomolecular Engineering, Yonsei University, Seoul, Republic of Korea; 2 Department of Radiology, College of Medicine, Yonsei University, Seoul, Republic of Korea; 3 Nanomedical National Core Research Center, Yonsei University, Seoul, Republic of Korea; 4 YUHS-KRIBB Medical Convergence Research Institute, Seoul, Republic of Korea; Niigata University Graduate School of Medical and Dental Sciences, JAPAN

## Abstract

**Objectives:**

To obtain compensatory ultra-short echo time (UTE) imaging and T2-weighted (T2W) imaging of Watanabe heritable hyperlipidemic (WHHL) rabbits following dextran-coated magnetic nanocluster (DMNC) injection for the effective *in vivo* detection of inflammatory vascular wall.

**Methods:**

Magnetic nanoparticle was synthesized by thermal decomposition and encapsulated with dextran to prepare DMNC. The contrast enhancement efficiency of DMNC was investigated using UTE (repetition time [TR] = 5.58 and TE = 0.07 ms) and T2W (TR = 4000 and TE = 60 ms) imaging sequences. To confirm the internalization of DMNC into macrophages, DMNC-treated macrophages were visualized by cellular transmission electron microscope (TEM) and magnetic resonance (MR) imaging. WHHL rabbits expressing macrophage-rich plaques were subjected to UTE and T2W imaging before and after intravenous DMNC (120 μmol Fe/kg) treatment. *Ex vivo* MR imaging of plaques and immunostaining studies were also performed.

**Results:**

Positive and negative contrast enhancement of DMNC solutions with increasing Fe concentrations were observed in UTE and T2W imaging, respectively. The relative signal intensities of the DMNC solution containing 2.9 mM Fe were calculated as 3.53 and 0.99 in UTE and T2W imaging, respectively. DMNC uptake into the macrophage cytoplasm was visualized by electron microscopy. Cellular MR imaging of DMNC-treated macrophages revealed relative signals of 3.00 in UTE imaging and 0.98 in T2W imaging. *In vivo* MR images revealed significant brightening and darkening of plaque areas in the WHHL rabbit 24 h after DMNC injection in UTE and T2W imaging, respectively. *Ex vivo* MR imaging results agreed with these *in vivo* MR imaging results. Histological analysis showed that DMNCs were localized to areas of inflammatory vascular wall.

**Conclusions:**

Using compensatory UTE and T2W imaging in conjunction with DMNC is an effective approach for the noninvasive *in vivo* imaging of atherosclerotic plaque.

## Introduction

Superparamagnetic nanoparticles have been widely applied as MR imaging contrast agents and molecular imaging probes combined with a targeting moiety in clinical studies, as in magnetic cell tracking with MR imaging, molecular imaging via MR imaging, and MR imaging-guided theragnosis.[1–6] In most cases, magnetic nanoparticles have been used as T2 shortening negative contrast agents in T2W imaging, but have rarely been used for T1 contrast enhancement because of the predominant spin diphase effect of magnetic nanoparticles.[7–9] A fundamental drawback of T2W imaging with negative contrast, however, is that the agent cannot be distinguished from other sources of signal loss in the image due to intrinsic signal voids, such as motion artifacts, hemorrhage, and organs with originally low background signals, such as lung (air) and lumen (blood). Additionally, accumulation of magnetic nanoparticles induces strong dephasing with image distortion, making accurate localization and quantitative imaging difficult.[10, 11]

UTE imaging, which involves positive contrast based on extremely short echo time, allowing for T1 signal acquisition with suppressed T2 decay from magnetic nanoparticles, can supplement the limitations of negative contrast imaging.[12, 13] In this study, we developed a dextran-coated magnetic nanocluster (DMNC) as a molecular imaging probe to enable the precise detection of macrophages expressing scavenger receptor A (SR-A) via compensatory UTE and T2W imaging. SR-A family is expressed on the cell surface of tissue macrophages, including macrophage foam cells, and have been detected on aortic endothelial cells and vascular smooth muscle cells within atherosclerotic plaque, thus SR-A are currently one of the most appealing targets at all stages of atherosclerosis.[14] In previous research, dextran and their derivatives (sulfated dextran, carboxyl dextran, and thiol-dextran) could be used for targeting SR-A, thus dextran layer on the surface of DMNC was designed for preferential uptake into the cytoplasm of macrophages through SR-A with highly biocompatible characteristics.[15–23] The magnetic nanoparticle cluster core of the DMNC was introduced as both a positive and negative contrast agent for UTE and T2W imaging. To assess the compensatory UTE and T2W imaging potential of DMNC, solution MR imaging and cellular MR imaging experiments were performed and *in vivo* MR imaging was conducted in WHHL rabbits as a chronic inflammation model with macrophage-initiated atherosclerotic plaques, following the intravenous injection of DMNC.[24–26] In atherosclerosis, macrophage accumulation leads to the formation of unstable plaques by inducing the production of various cytokines and chemokines, and may result in sudden death due to the rupture of the thrombus.[27–29] Compensatory UTE and T2W imaging of macrophages based on DMNC should overcome the limitations of a single negative contrast imaging sequence, provide accurate diagnoses, and facilitate the pathological investigation of atherosclerosis.

## Methods

### Dextran-coated Magnetic Nanocluster (DMNC)

For the synthesis of DMNC, the details of the compound are descibed here. Dextran T-10 (Mw: 10,000 Da) was obtained from Pharmacia Biotech. 1-Pyrenebutyric acid, 1,3-dicyclohexylcarbodiimide, 4-dimethylaminopyridine, anhydrous dimethyl sulfoxide, triethylamine, iron(III) acetylacetonate, 1,2-hexadecanediol, oleic acid, oleylamine, and benzyl ether were purchased from Sigma-Aldrich. Centrifugal filters (Amicon Ultra, 30,000 MWCO cut-off) were purchased from Millipore. Hydrophilic syringe filters with 0.45 μm pore size (DISMIC, PTFE 25HP045AN) were purchased from ADVANTEC. All other chemicals and reagents were of analytical grade and obtained from Sigma-Aldrich.

DMNC was synthesized as described in our previous studies. We synthesized monodisperse 12-nm magnetic nanoparticle (MNP) by seed-mediated growth through the thermal-decomposition method. Pyrenyl dextran (Pydex), amphiphilic polymer was synthesized for the immobilization of the hydrophobic surface of MNP. To synthesize Pydex, the hydroxyl group of dextran was conjugated with the carboxylic acid of 1-pyrenebutyric acid through the esterification reaction. To prepare DMNC, MNP was then clustered and coated with Pydex through the nanoemulsion method.

The size and morphology of DMNC were investigated using transmission electron microscopy (TEM, JEM-2100 LAB6, JEOL Ltd.). The hydrodynamic diameter and surface charge of DMNC were measured using laser scattering (ELS-Z, Otsuka Electronics). The magnetic hysteresis loop and the saturation magnetization of DMNC were determined in dried samples at room temperature using a vibrating sample magnetometer (Model-7300, Lakeshore).

### Solution MR Imaging of DMNC

Solution MR imaging experiments were performed using a SIEMENS 3.0 T MR imaging system (MAGNETOM Trio, SIEMENS) with an 8-channel wrist coil using the UTE and T2W imaging sequences. The sequence parameters for UTE imaging were TR = 5.58 ms, TE = 0.07 ms, flip angle = 20°, field of view: 200 × 100 mm^2^, voxel size = 0.5 × 0.5 × 1 mm^3^, and number of acquisitions = 1, and the sequence parameters for T2W imaging were TR = 4,000 ms, TE = 60 ms, flip angle = 20°, field of view: 200 × 100 mm^2^, voxel size = 0.5 × 0.5 × 1 mm^3^, and number of acquisitions = 1. Tubes containing the DMNC solution with various Fe concentrations were mounted in a sample holder and located at the iso-center of the magnet for MR imaging.

To measure the DMNC signal intensity, circular regions of interest (ROI) were placed on individual images of each solution sample. After measuring the signal intensities of each sample and water, the relative signal intensity was calculated as follows: │I—I_water_│/I_water_ where I and I_water_ are the signal intensities of the selected DMNC sample and water, respectively. The relative signal intensity was then plotted versus Fe concentration.

### *In vitro* experiments

RAW264.7 cells (8.0 × 10^8^ cells) were implanted in a Petri dish at 37°C overnight and washed three times using phosphate-buffered saline (PBS, pH 7.4). The cells were then treated with DMNC (20 μg Fe) for 24 h. Subsequently, the cells were washed with PBS three times to eliminate unbound DMNC, detached using a cell scraper, and collected and re-suspended in 200 μL of fixation solution.[30–32] Cellular internalization was verified by MR imaging and transmission electron microscopy (TEM, JEOL-1100). *In vitro* MR imaging experiments followed the same procedure as that used for MR imaging of the DMNC solution.

### Animal preparation

WHHL rabbits were supported by the Cardiovascular Product Evaluation Center (CPEC) in the Yonsei University Health System, Korea (www.cpec.co). Experiments were conducted in 10-month-old male WHHL rabbits with body weights of 3.0±0.4 kg. At this age, Watanabe rabbits exhibit active plaque formation within their aortic walls.[24, 26] Ten-month-old male New Zealand White rabbits with, body weights of 2.8±0.5 kg were purchased from DooYeol Biotech, Korea (www.dybiotech.co.kr) and used as normal controls. All rabbits survived until subsequent sacrifice without any clinical signs of respiratory or cardiac failure during the study. All animal experiments were conducted with the approval of the Institutional Animal Care and Use Committee Yonsei University Health System (Project No. 2011–0094).

### Animal MR imaging experiment

*In vivo* MR imaging experiments were performed with a SIEMENS 3.0 T MR imaging system (MAGNETOM Trio, SIEMENS) with an 8-channel knee coil using the UTE and T2W imaging sequences. *In vivo* MR imaging of the rabbit aorta was performed before and after the administration of DMNC into the rabbit ear vein using a syringe (120 μmol Fe/kg). Specifically, animals were imaged prior to DMNC injection (0 h), and then imaging was repeated at 0.25 h (immediate), 2 h on day 0, and 24 h on day 1 following DMNC injection. After *in vivo* MR imaging, the rabbit was subsequently sacrificed with an overdose of thiopental, and the rabbit aorta was extracted immediately. Subsequently, *ex vivo* MR imaging of aortic wall was performed. Animal UTE images were obtained using the following sequence parameters: TR = 5.58 ms, TE = 0.07 ms, flip angle = 20°, field of view: 200 × 100 mm^2^, voxel size = 0.5 × 0.5 × 1 mm^3^, and number of acquisitions = 1. For animal T2W imaging, the following sequence parameters were adopted: TR = 4,000 ms, TE = 60 ms, flip angle = 20°, field of view: 200 × 100 mm^2^, voxel size = 0.5 × 0.5 × 1 mm^3^, and number of acquisitions = 1. Fifty transverse (thoracic aorta) and 50 (aortic arch) slices were acquired from both sequences.

### Data analysis of MR imaging

All the animal MR imaging data were transferred from the MR imaging scanner to a Dicom image server for quantitative analysis, and then the aortic wall area and the average signal intensity were analyzed by Centricity IT software solutions (GE Healthcare). For the quantification of intravascular contrast changes following DMNC injection, the signal intensity in the lumen was calculated and plotted versus time.[26, 33]

Intravascular MR signal change after DMNC injection: For the quantification of signal intensity in the lumen, regions of interest (ROI) were placed manually in the lumen of the aortic arch and thoracic aorta. The signal intensity in the lumen was then plotted at various time intervals after DMNC injection.

Contrast enhancement of the atherosclerotic plaque: Images were viewed with magnification and pre- and post-DMNC injection images from any individual were adjusted to ensure identical window/level settings. To allow exact matching between pre- and post-DMNC injection images, anatomical landmarks (position of the aortic arch, renal arteries, and iliac bifurcation) were used to guide ROI positioning. The presence of DMNC within the plaque was confirmed by noting whether the matched post-injection image contained a new region(s) of low (for T2W images)/high (for UTE images) signal intensity within the vessel wall. On the pre-DMNC injection image, the ring-shaped ROI was defined to include whole region of vessel and vessel wall except lumen. The delineated ROI was then copied and transposed to the same location on the post-DMNC injection image to provide a “mirror” location for comparative analysis. The signal intensity of these ROI was then converted to color coded image.

Statistical analysis: Statistical evaluation of data was performed using the analysis of variance test and Student’s t-test. A p-value less than 0.01 was considered statistically significant.

### Histological analysis

For the histological analysis, the entire aorta from the aortic root to below the iliac bifurcation was harvested. The aorta was stained with: 1) Oil Red O (ORO) for specific lipid staining; 2) Hematoxylin and eosin (H&E) with Prussian blue (PB) staining for iron staining.

ORO staining: 30 mL ORO stock solution (0.5 g/100 mL isopropanol) was mixed with 20 mL distilled water to prepare the ORO working solution. Extracted rabbit aorta was rinsed with 60% isopropanol and incubated with ORO working solution. After 15 min, the aorta was rinsed with 60% isopropanol and distilled water to remove unabsorbed ORO.

H&E with PB staining: Specimens were dehydrated using ethanol and cleared with xylene. Slices were mounted onto glass slides and soaked twice in a container filled with hematoxylin for 10 min to stain nuclei, followed by rinsing with deionized water. The cytoplasm was counter-stained with eosin and dehydrated in the same manner as mentioned earlier. Subsequently, the specimens were immersed in iron staining solution (20% hydrochloric acid: potassium ferrocyanate = 1:1) for 30 min at room temperature to stain iron content in tissues. Then the samples were rinsed in deionized water three times to remove residual staining solution.

All stained aorta from DMNC-treated WHHL rabbits (Experiment) were visualized using a virtual microscope (Olympus BX51, Japan) and Olyvia software.[14, 34]

## Results

### UTE and T2W imaging of DMNC solution

**Fig 1:**
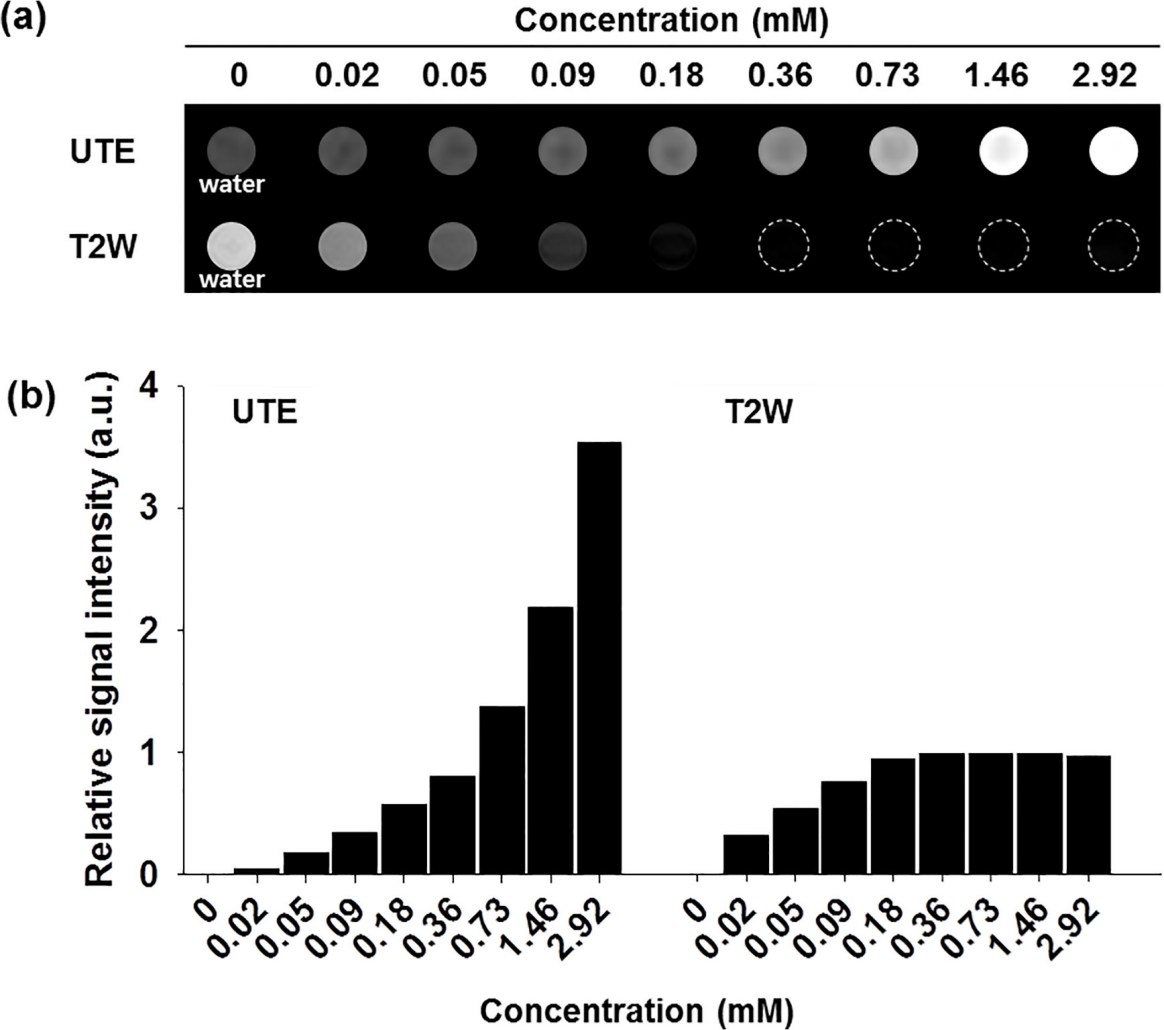
Solution MR imaging of DMNC. (a) UTE and T2W images of DMNC solution, and (b) relative signal intensity versus Fe concentration.

To obtain compensatory UTE and T2W imaging, DMNC as a molecular imaging probe was prepared and showed uniform and spherical shape with highly water-stability for 15 day (S1 Fig). To investigate the compensatory contrast enhancement effect, DMNC solutions containing various Fe concentrations were visualized by UTE and T2W imaging (Fig 1A). Positive contrast enhancement was obtained in all DMNC samples using the UTE sequence. Recognizable brightening without a contrast void occurred, followed by an increase in Fe concentrations in all selected concentration ranges. In T2W imaging, a negative contrast effect was observed in the DMNC solution. The solution samples were visibly darker until the Fe concentration reached 0.18 mM. However, the contrast void was observed at Fe concentrations of 0.36 mM and greater. The signal intensities of the DMNC solutions in UTE and T2W images are provided in S1 Table and agreed with the imaging results.

To quantify the contrast enhancement effect of DMNC, the relative signal intensity (arbitrary unit, a.u.) was calculated from each DMNC solution using the water signal intensity as the baseline reference, and then plotted versus Fe concentration (Fig 1B).[26] At Fe concentrations ranging from 0 to 0.18 mM, a significant increase in the relative signal intensity was observed in T2W images (0 to 0.94) compared with UTE images (0 to 0.57). At Fe concentrations over 0.18 mM, the relative signal intensity of DMNC in UTE images increased continuously without signal saturation until the highest concentration used (3.53 at 2.92 mM). However, no further increase in the saturated signal was observed in T2W images (0.99 at 2.92 mM) due to signal voids.[12]

### *In vitro* treatment of macrophages with DMNC

**Fig 2:**
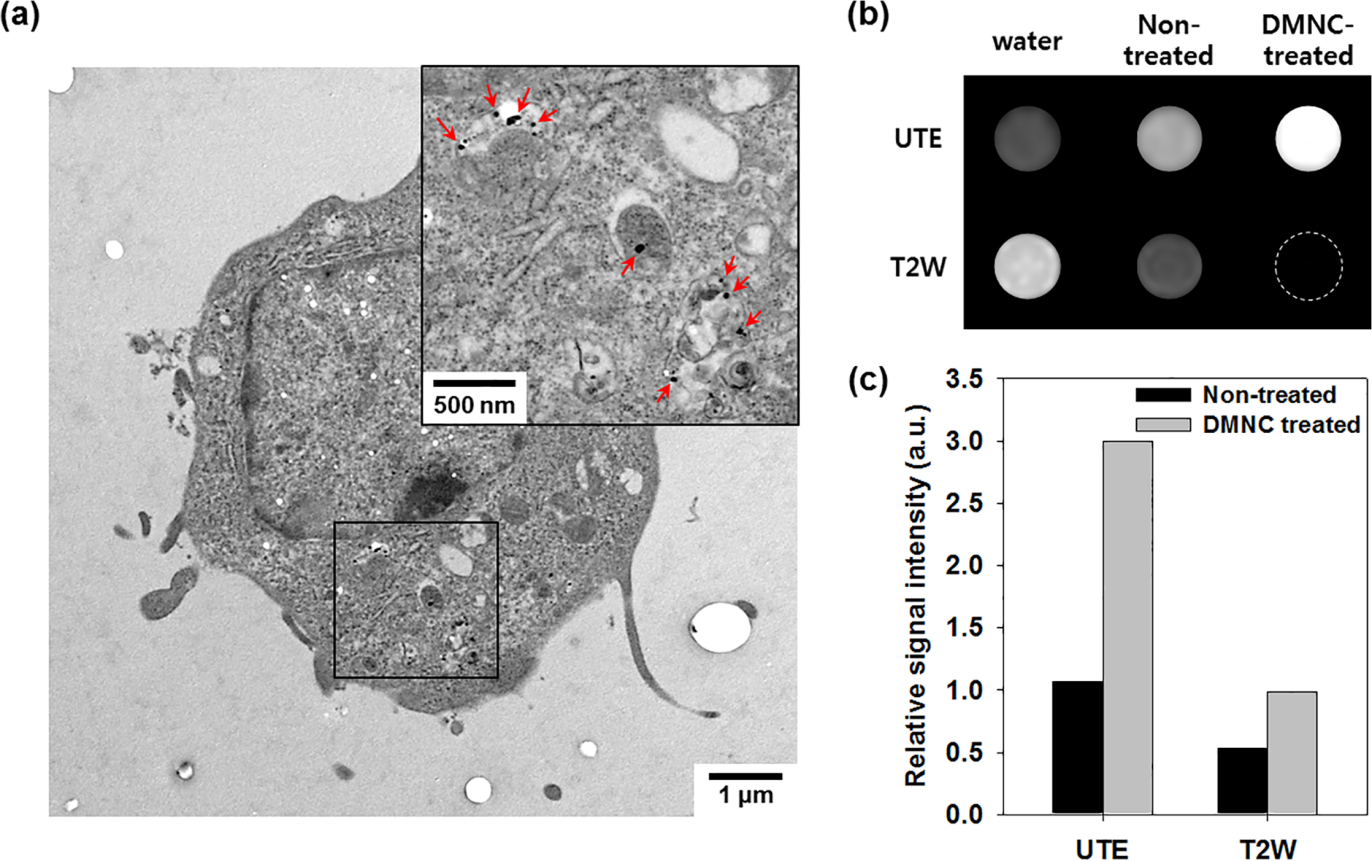
*In vitro* treatment of macrophages with DMNC. (a) TEM image of macrophage treated with DMNC (inset: magnified image of selected area). (b) UTE and T2W images and (c) relative signal intensities of macrophages following DMNC treatment (20 μg Fe).

As shown in Fig 2, DMNC-treated macrophages (RAW264.7 cells) were visualized by cellular TEM and MR imaging to confirm the uptake of DMNC into macrophages and to demonstrate the cellular MR imaging ability of DMNC.[16] As seen in cellular TEM images (Fig 2A), a considerable amount of DMNCs (red arrow) were located in the cytoplasm of macrophages, and the cellular structures were sustained without damage. In MR imaging of macrophages treated with DMNC, UTE images exhibited positive contrast enhancement and T2W images showed negative contrast enhancement, compared to non-treated cells (Fig 2B). To precisely quantify the contrast enhancement effect of DMNC, the relative signal intensity was calculated and is presented in Fig 2C. In UTE imaging, the relative signal intensity of macrophages treated with DMNC showed a significant signal increase (3.00) compared with non-treated cells (1.07). On the other hand, in T2W imaging, only a slight increase in the relative signal intensity was observed in DMNC-treated cells (0.98) compared with non-treated cells (0.54), because of the contrast void.

### Characterization of atherosclerotic plaques

**Fig 3:**
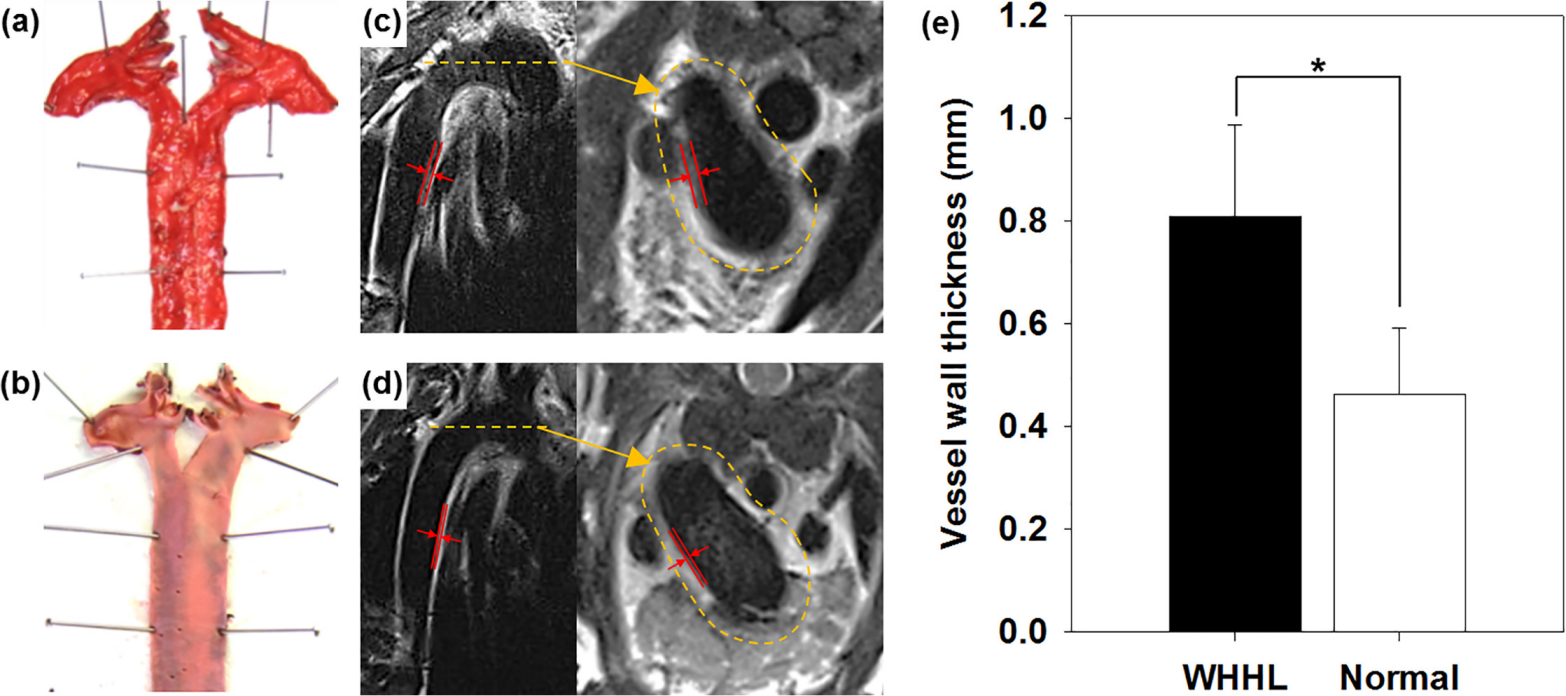
Characterization of atherosclerotic plaques. ORO staining of aorta of (a) WHHL rabbits and (b) normal rabbits. The aortic arch and thoracic aorta of (c) WHHL rabbit and (d) normal rabbit were visualized by T2W imaging. (e) The vessel wall thickness of WHHL and normal rabbits were measured from the MR imaging results (*p<0.0001).

To demonstrate the *in vivo* MR imaging ability of DMNC based on compensatory UTE and T2W imaging, the WHHL rabbit was chosen as a macrophage-induced atherosclerosis model that shows plaque formation in its vessel wall.[24] To confirm the presence of atherosclerotic lesions, ORO immunostaining was performed in WHHL and normal rabbits. WHHL rabbits exhibited lipid-rich plaque formation with a completely red-stained aortic wall (Fig 3A), in contrast to normal rabbits (Fig 3B) Wall thickening was observable in the WHHL rabbit (Fig 3C) from representative baseline T2W images of the aortic arch and the thoracic aorta, in contrast to normal rabbits (Fig 3D). The quantified wall thickness was 0.81±0.18 mm in the WHHL rabbit and 0.46±0.13 mm in the normal rabbit (p<0.001, Fig 3E).[26]

### Intraluminal MR imaging of WHHL rabbit after DMNC injection

**Fig 4:**
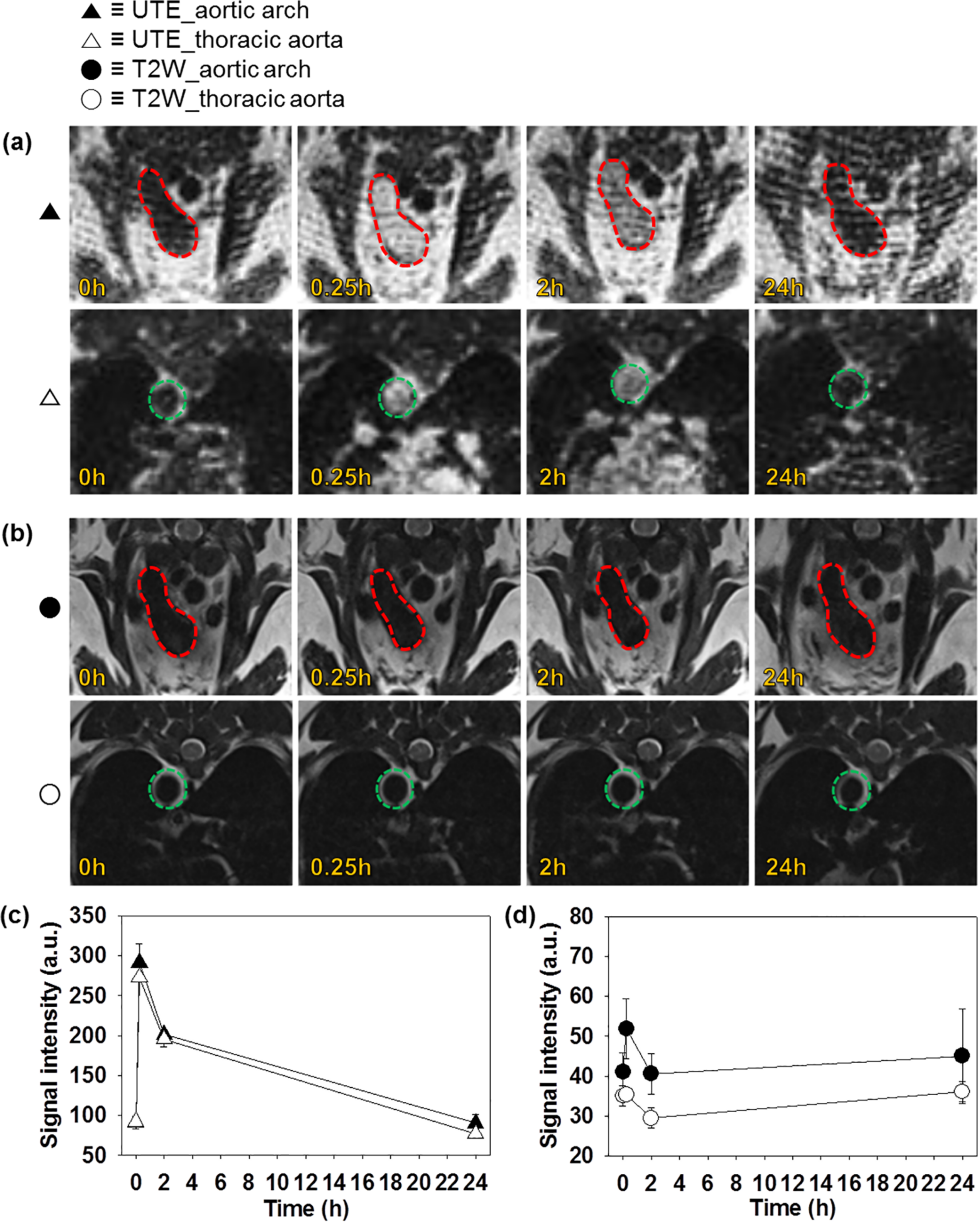
Intravascular MR imaging of WHHL rabbit after DMNC injection. Aortic arch (red) and thoracic aorta (green) at 0, 0.25, 2, and 25 h after DMNC injection (0 h: baseline without DMNC injection) were visualized by (a) UTE and (b) T2W imaging. The intravascular signal intensity of (c) UTE and (d) T2W imaging was quantified and plotted versus time.

As shown in Fig 4, compensatory UTE and T2W imaging of the aortic arch and thoracic aorta were performed after intravenous injection of DMNC, and the signal intensity in the lumen was calculated to investigate the intraluminal kinetics of DMNC in WHHL rabbits (▲, UTE_aortic arch; △, UTE_thoracic aorta; ●, T2W_aortic arch; ○, T2W_thoracic aorta).[35] In Fig 4A, UTE images of the lumen appeared characteristically dark (0 h) prior to treatment with DMNC. Following DMNC injection, the lumen images brightened, reflecting the contribution of DMNC to strong intravascular signal enhancement in the blood pool (0.25 h). Brightening remained at 2 h and recovered by 24 h, because DMNC was removed from blood pool over time due to excretion. However, no remarkable contrast change was found in T2W imaging results of the aorta, because the darkening effect of DMNC in T2W imaging was not distinctive in the lumen, the background image of which is originally dark (Fig 4B).

Further, the signal intensity was measured in the intraluminal region and agreed with the MR imaging results. The signal intensity obtained from UTE images showed that the signal intensity significantly increased immediately following DMNC injection (▲: 91.3±7.7 to 291.6±23.6, △: 92.1±4.7 to 272.6±3.3), and decreased at 2 h (▲: 201.4±3.0, △: 195.4±9.5), reverting to baseline values by 24 h (▲: 90.5±10.2, △: 77.2±2.4) (Fig 4C). In the case of T2W imaging, however, only a slight signal change was observed in the lumen (●: 41.0±4.9 and ○: 35.1±2.5 at 0 h, ●: 51.9±7.5 and ○: 35.3±1.6 at 0.25 h, ●: 40.6±5.1 and ○: 29.5±2.5 at 2 h, ●: 45.3±11.8 and ○: 36.1±2.6 at 24 h) (Fig 4D).

### *In vivo* MR imaging of WHHL rabbit aorta after DMNC injection

**Fig 5:**
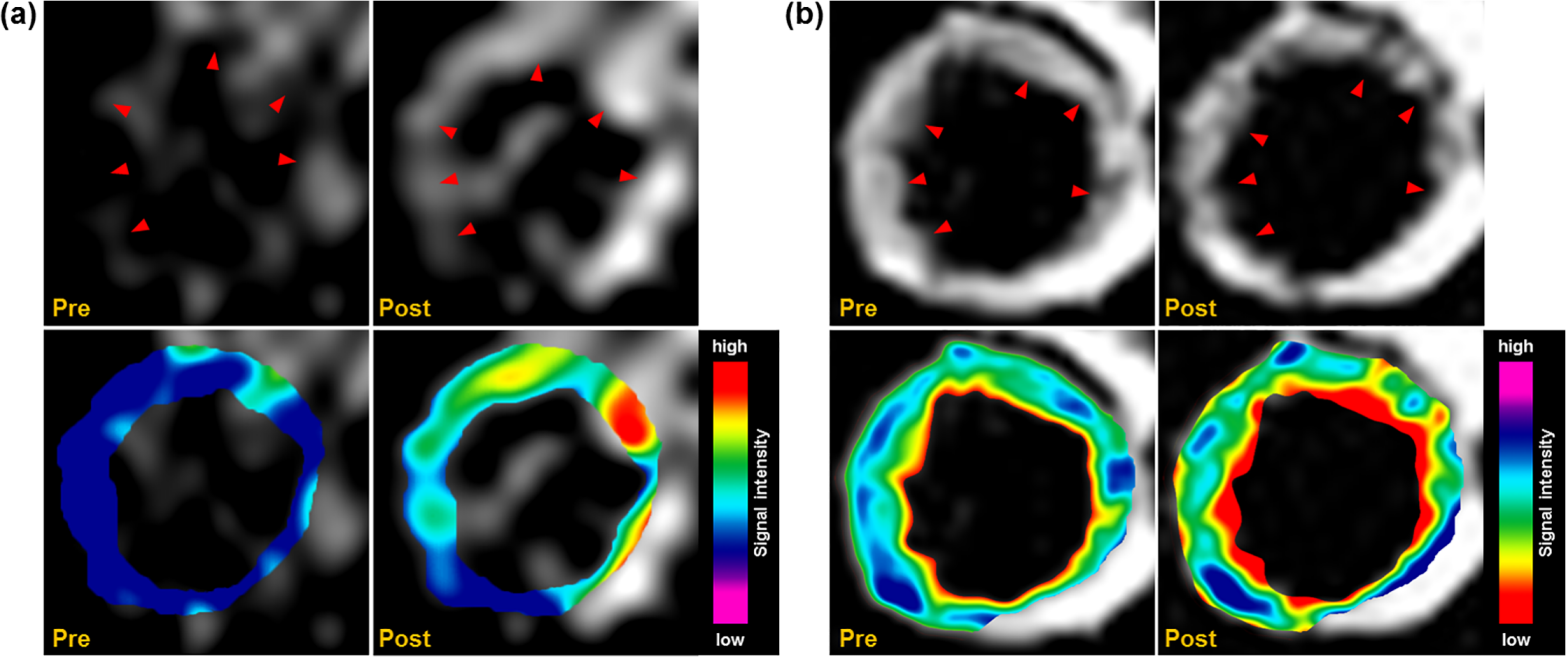
*In vivo* MR imaging of WHHL rabbit aorta after DMNC injection. The thoracic aorta of the WHHL rabbit was visualized by (a) UTE and (b) T2W imaging after DMNC treatment, and their color-coded images are presented (bottom row). Red arrows indicates contrast-enhanced regions.

To demonstrate the *in vivo* MR imaging ability of DMNC, the thoracic aorta in the WHHL rabbit was visualized by compensatory UTE and T2W imaging after intravenous injection of DMNC, as shown in Fig 5. On UTE imaging (Fig 5A), the vessel wall was homogeneously suppressed, appearing a characteristic gray prior to DMNC injection (pre, upper row), while a striking brightening (positive contrast) was seen following DMNC injection (red arrows in post, upper row) because of DMNC deposition in the vessel wall. Upon T2W imaging (Fig 5B), subtle spots of distortion (negative contrast) were detected in the aorta after DMNC injection (red arrows in post, upper row) compared with baseline images (pre, upper row). Color-coded images of the vessel wall indicate that overall signal increase appeared in the whole vessel wall as seen that dark blue-purple are changed to red-green in UTE images (bottom row, Fig 5A). In T2W images, some spots of region which is located on the border between vessel wall and lumen are changed to red (bottom row, Fig 5B).

### Histological investigation of WHHL rabbit aorta

**Fig 6:**
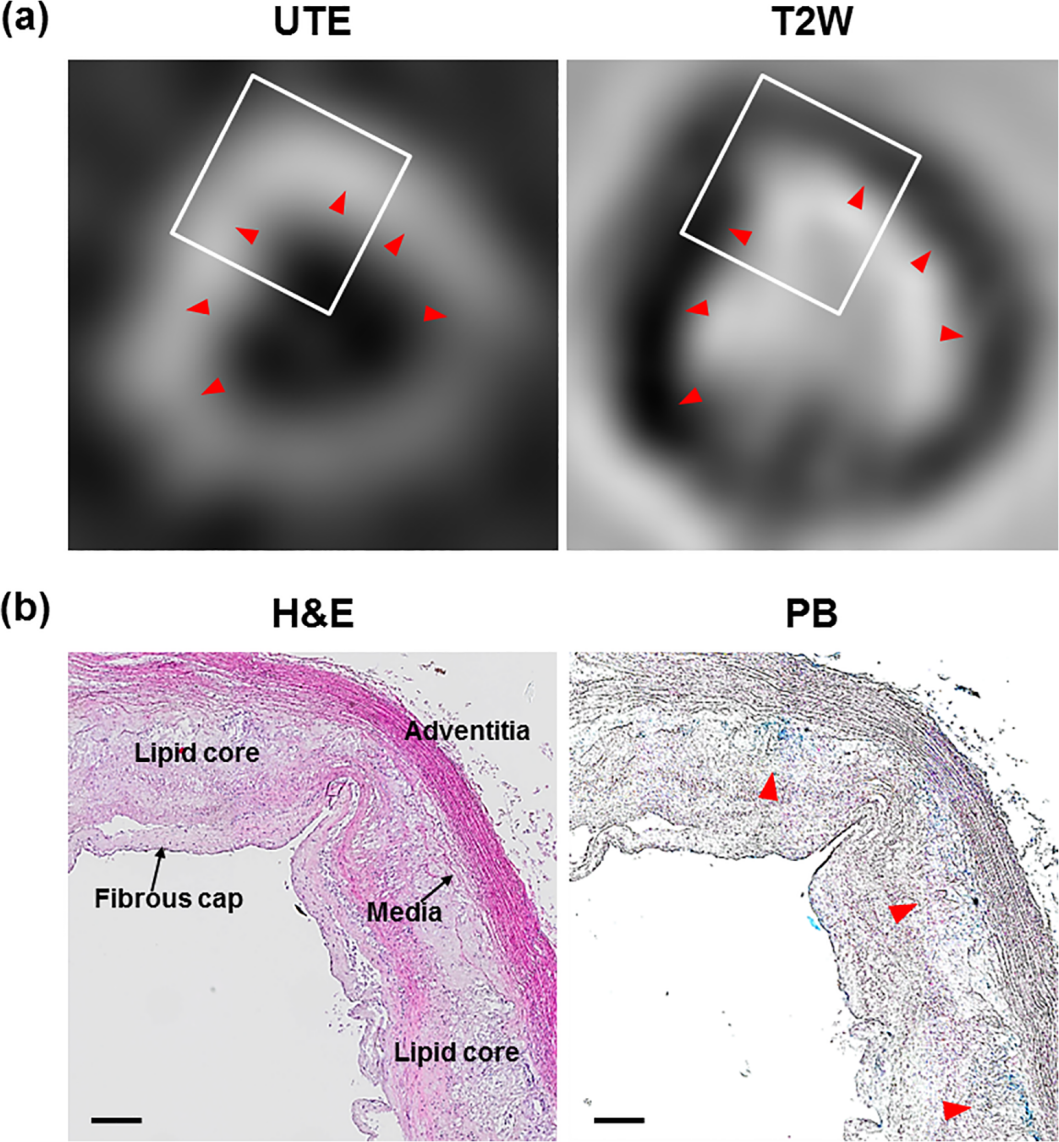
Extracted rabbit aorta was investigated by MR imaging and histological staining. (a) Extracted aorta was visualized by UTE and T2W imaging. (b) Histological investigation was performed by H&E, PB staining. The rectangle in (a) the *ex vivo* MR Imaging indicates the area shown in (b).

*Ex vivo* MR imaging and histological analysis of the extracted WHHL rabbit aorta was performed to confirm the delivery of DMNC (Fig 6). In Fig 6A, *ex vivo* MR imaging of the extracted WHHL rabbit aorta treated with DMNC showed strong contrast enhancement in the vessel wall in UTE and T2W images. Fig 6B shows an H&E stained aorta revealing the basic morphology of the plaque lesion with a lipid core and macrophages deposition. The blue dots within the plaque lesion, detected by PB staining (red arrow), indicate the presence of iron from the DMNC.

## Discussion

### Advantageous features of compensatory UTE/T2W imaging based on DMNC

The advance in this study is that DMNC for MR imaging of atherosclerotic plaque was initially designed to optimize the structure and properties from nano-scale for sensitive detection of atherosclerotic plaque. 1) Thermal-decomposition method to synthesize MNP improved contrast effect of MNP based on highly crystalline nano-structure.[2, 36] 2) Artificially fabricated Pydex copolymer could provide macrophage-targetable moiety with suitable amphiphilic coating agent for hydrophobic MNP. 3) Nanoemulsion of MNP with Pydex fabricated dextran-coated magnetic core consisted of clustered MNP which exhibited better contrast enhancement effect than single MNP at same concentration.[37]

To supplement the typical drawback of T2W imaging, UTE imaging, which could enhance the T1 effect of the magnetic nanoparticles, was suggested as a positive contrast imaging technique. To capture the T1 enhancement effect from the magnetic nanoparticle, UTE imaging uses rapid transmit/receive switching, radial mapping of k-space, and a very short non-selective RF excitation of 60 μs that allows signal acquisition to start almost immediately after RF excitation.[12] With respect to the solution MR imaging of DMNC (Fig 1), UTE images showed the advantages of continuous brightening with positive contrast on a dark background without a contrast void. However, the T2W imaging result represents the limitation of the contrast void with indistinguishable images on a dark background. Although the relative signal intensity of the UTE image (3.53) was 3.6 times higher than the relative signal intensity of the T2W image (0.99) at the maximum Fe concentration (2.92 mM) of the DMNC solution, the relative signal intensity at 0.18 mM Fe increased more sensitively, from 0 to 0.94, using T2W imaging as compared to UTE imaging, which showed a change from 0 to 0.57. From the comparison of the UTE and T2 imaging results of DMNC solution, we conclude that T2W shows sensitive contrast and signal changes at low Fe concentrations, but that UTE imaging is the alternative solution for contrast void and signal saturation of T2W imaging at high Fe concentrations.

### Cellular imaging ability of DMNC via UTE/T2W imaging

Macrophages influence the clinical outcomes of various inflammatory diseases, including atherosclerosis. Therefore, macrophage detection should help identify patients with subclinical inflamed lesions, and provide new and important insights for preventive cardiovascular medicine.[29]

The presented cellular TEM image in Fig 2A shows that macrophages internalize DMNC to a large extent without damaging cellular structures. The underlying mechanisms of nanoparticle uptake by macrophages remain incompletely understood, but a recent study has suggested an interaction between macrophage SR-A and dextran.[38] To prove the biomedical utility of compensatory UTE/T2W imaging using DMNC, cellular MR imaging of macrophages treated with DMNC was performed (Fig 2B). UTE imaging of DMNC-laden macrophages exhibited remarkably brightened contrast enhancement, whereas T2W imaging showed darkening with a contrast void. Additionally, the relative signal of the UTE image (3.00) was 3.1 times higher than that of the T2W image (0.98). These cellular MR imaging results indicate that UTE imaging combined with DMNC is highly acceptable for sensitive macrophage detection, and can be used in various *in vitro* MR imaging studies.

### *In vivo* detection of macrophages with compensatory UTE/T2W imaging using DMNC

To investigate the *in vivo* macrophage detection ability of DMNC via compensatory UTE/T2W imaging, the WHHL rabbit, which develops macrophage-rich atherosclerotic plaques, was chosen as the model of chronic inflammation. WHHL rabbits exhibit increased wall thickness compared to normal rabbits, and show pronounced intimal thickening (0.35 mm thicker than normal rabbit), associated with lipid- and macrophage-rich plaque formation (Fig 3).[24, 26, 28]

Before the detection of atherosclerotic plaques, the intraluminal kinetics of DMNC was analyzed based on intravascular contrast and signal change (Fig 4). Based on quantitative analysis of the intraluminal signal, we observed that the signal intensity of blood at 24 h on day 1 approached the signal intensity of blood at baseline (0 h), which indicated complete clearance of the agent from the blood pool at this point. The clearance of DMNC in the blood pool provided better contrast between the lumen and DMNC-deposited macrophages on the aortic wall. Therefore, the optimal time for effective analysis of *in vivo* aortic wall MR images was 24 h after DMNC administration. Furthermore, UTE imaging was found to be a better technique than T2W imaging for studying intraluminal DMNC kinetics, because UTE imaging provides a sensitive signal change (200.3 = 291.6–91.3) with an accurately distinguishable intraluminal contrast change, whereas T2W imaging shows a slight signal change (10.9 = 51.9–41.0) with no remarkable intraluminal contrast change.

Using DMNC with compensatory UTE/T2W imaging, striking contrast enhancement could be readily detected in areas containing macrophage-rich plaques at 24 h (Fig 5).[26] In UTE imaging, the increase in positive signal correlated with DMNC-laden macrophage density in plaque areas and allowed for the highly sensitive and specific detection of macrophages. From T2W imaging, negative contrast could be detected in the wall of the aorta, which was not present at baseline. T2W imaging could provide better resolution of *in vivo* MR images of the aorta than seen with UTE imaging, although subtle spots of negative contrast could not be exclusively attributed to DMNC deposition, due to signal voids caused by respiratory motion artifacts or the absence of tissue in the same area. For the better understanding of imaging results, subtracted images for each UTE and T2W are presented in S2 Fig These positive and negative signals on the *in vivo* MR images corresponded to the deposition of DMNC in macrophage-rich atherosclerotic plaques, as seen on *ex vivo* MR imaging results and histological analysis (Fig 6).

## Conclusion

In this study, UTE/T2W imaging was combined with macrophage-targetable DMNC to generate compensatory positive and negative contrast images of atherosclerotic plaques. Using this compensatory MR imaging technique, the intraluminal kinetics of DMNC was investigated and areas containing macrophage-rich plaques could be highlighted. UTE imaging with positive contrast effectively supplemented the typical weaknesses of T2W imaging: contrast void and signal saturation. From this result, DMNC enabled specifically targeted atherosclerosis MR imaging and the presented compensatory imaging technique should provide non-invasive evaluation of inflammatory vascular wall, and useful to monitor therapeutic interventions of atherosclerosis.

## Supporting Information

S1 Fig Characterization of DMNC. (a) TEM image of DMNC. (b) Size and surface charge variation of DMNC over 15 days. (c) R2 graph of DMNC from solution MR imaging at various Fe concentrations.
(TIF)

S2 Fig Subtracted in vivo MR imaging of WHHL rabbit after DMNC injection. Subtracted images of thoracic aorta using (a) T2W imaging (Sub = Pre—Post) and (b) UTE imaging (Sub = Post—Pre).
(TIF)

S1 Supporting Text Supporting file in text. (DOCX)

S1 Table Signal intensities and relative signal intensities of DMNC solutions. (TIF)

## References

[1] Modo M, Cash D, Mellodew K, Williams SC, Fraser SE, Meade TJ, et al Tracking Transplanted Stem Cell Migration Using Bifunctional, Contrast Agent-Enhanced, Magnetic Resonance Imaging. NeuroImage. 2002;17(2):803–11. 12377155

[2] Lee J-H, Huh Y-M, Jun Y-w, Seo J-w, Jang J-t, Song H-T, et al Artificially engineered magnetic nanoparticles for ultra-sensitive molecular imaging. Nat Med. 2007;13(1):95–9. 17187073 10.1038/nm1467

[3] Lim E-K, Huh Y-M, Yang J, Lee K, Suh J-S, Haam S. pH-Triggered Drug-Releasing Magnetic Nanoparticles for Cancer Therapy Guided by Molecular Imaging by MRI. Advanced Materials. 2011;23(21):2436–42. 10.1002/adma.201100351 21491515

[4] Flacke S, Fischer S, Scott MJ, Fuhrhop RJ, Allen JS, McLean M, et al Novel MRI Contrast Agent for Molecular Imaging of Fibrin: Implications for Detecting Vulnerable Plaques. Circulation. 2001;104(11):1280–5. 11551880 10.1161/hc3601.094303

[5] Chertok B, Moffat BA, David AE, Yu F, Bergemann C, Ross BD, et al Iron oxide nanoparticles as a drug delivery vehicle for MRI monitored magnetic targeting of brain tumors. Biomaterials. 2008;29(4):487–96. 17964647 10.1016/j.biomaterials.2007.08.050 PMC2761681

[6] Kim B, Yang J, Hwang M, Choi J, Kim H-O, Jang E, et al Aptamer-modified magnetic nanoprobe for molecular MR imaging of VEGFR2 on angiogenic vasculature. Nanoscale Research Letters. 2013;8(1):1–10. 10.1186/1556-276X-8-1 24066922 PMC3849016

[7] Weissleder R, Elizondo G, Wittenberg J, Lee AS, Josephson L, Brady TJ. Ultrasmall superparamagnetic iron oxide: an intravenous contrast agent for assessing lymph nodes with MR imaging. Radiology. 1990;175(2):494–8. 2326475 10.1148/radiology.175.2.2326475

[8] Wang Y-X, Hussain S, Krestin G. Superparamagnetic iron oxide contrast agents: physicochemical characteristics and applications in MR imaging. European Radiology. 2001;11(11):2319–31. 11702180 10.1007/s003300100908

[9] Simon G, Bauer J, Saborovski O, Fu Y, Corot C, Wendland MF, et al T1 and T2 relaxivity of intracellular and extracellular USPIO at 1.5T and 3T clinical MR scanning. European Radiology. 2006;16(3):738–45. 16308692 10.1007/s00330-005-0031-2

[10] Stuber M, Gilson WD, Schär M, Kedziorek DA, Hofmann LV, Shah S, et al Positive contrast visualization of iron oxide-labeled stem cells using inversion-recovery with ON-resonant water suppression (IRON). Magnetic Resonance in Medicine. 2007;58(5):1072–7. 17969120 10.1002/mrm.21399

[11] Shapiro EM, Skrtic S, Koretsky AP. Sizing it up: Cellular MRI using micron-sized iron oxide particles. Magnetic Resonance in Medicine. 2005;53(2):329–38. 15678543 10.1002/mrm.20342

[12] Zhang L, Zhong X, Wang L, Chen H, Wang YA, Yeh J, et al T1-weighted ultrashort echo time method for positive contrast imaging of magnetic nanoparticles and cancer cells bound with the targeted nanoparticles. Journal of Magnetic Resonance Imaging. 2011;33(1):194–202. 10.1002/jmri.22412 21182139 PMC3785614

[13] Girard OM, Du J, Agemy L, Sugahara KN, Kotamraju VR, Ruoslahti E, et al Optimization of iron oxide nanoparticle detection using ultrashort echo time pulse sequences: Comparison of T1, T2*, and synergistic T1 − T2* contrast mechanisms. Magnetic Resonance in Medicine. 2011;65(6):1649–60. 10.1002/mrm.22755 21305596 PMC3097261

[14] Daugherty A, Manning MW, Cassis LA. Angiotensin II promotes atherosclerotic lesions and aneurysms in apolipoprotein E–deficient mice. The Journal of Clinical Investigation. 2000;105(11):1605–12. 10841519 10.1172/JCI7818 PMC300846

[15] Nahrendorf M, Zhang H, Hembrador S, Panizzi P, Sosnovik DE, Aikawa E, et al Nanoparticle PET-CT Imaging of Macrophages in Inflammatory Atherosclerosis. Circulation. 2008;117(3):379–87. 18158358 10.1161/CIRCULATIONAHA.107.741181 PMC2663426

[16] Lim E-K, Jang E, Kim B, Choi J, Lee K, Suh J-S, et al Dextran-coated magnetic nanoclusters as highly sensitive contrast agents for magnetic resonance imaging of inflammatory macrophages. Journal of Materials Chemistry. 2011;21(33):12473–8.

[17] Jaffer FA, Nahrendorf M, Sosnovik D, Kelly KA, Aikawa E, Weissleder R. Cellular imaging of inflammation in atherosclerosis using magnetofluorescent nanomaterials. Molecular Imaging. 2006;5(2):85–92. 16954022

[18] Kooi ME, Cappendijk VC, Cleutjens KB, Kessels AG, Kitslaar PJ, Borgers M, et al Accumulation of ultrasmall superparamagnetic particles of iron oxide in human atherosclerotic plaques can be detected by in vivo magnetic resonance imaging. Circulation. 2003;107(19):2453–8. 12719280 10.1161/01.CIR.0000068315.98705.CC

[19] You DG, Saravanakumar G, Son S, Han HS, Heo R, Kim K, et al Dextran sulfate-coated superparamagnetic iron oxide nanoparticles as a contrast agent for atherosclerosis imaging. Carbohydr Polym. 2014;101:1225–33. 10.1016/j.carbpol.2013.10.068 24299895

[20] Platt N, Gordon S. Is the class A macrophage scavenger receptor (SR-A) multifunctional?—The mouse's tale. J Clin Invest. 2001;108(5):649–54. 11544267 10.1172/JCI13903 PMC209390

[21] Jaffer FA, Libby P, Weissleder R. Molecular and cellular imaging of atherosclerosis: emerging applications. J Am Coll Cardiol. 2006;47(7):1328–38. 16580517 10.1016/j.jacc.2006.01.029

[22] Jarrett BR, Frendo M, Vogan J, Louie AY. Size-controlled synthesis of dextran sulfate coated iron oxide nanoparticles for magnetic resonance imaging. Nanotechnology. 2007;18(3):035603 10.1088/0957-4484/18/3/035603 19636126

[23] Choi R, Yang J, Choi J, Lim E-K, Kim E, Suh J-S, et al Thiolated Dextran-Coated Gold Nanorods for Photothermal Ablation of Inflammatory Macrophages. Langmuir. 2010;26(22):17520–7. 10.1021/la1029728 20929199

[24] Shiomi M, Ito T. The Watanabe heritable hyperlipidemic (WHHL) rabbit, its characteristics and history of development: A tribute to the late Dr. Yoshio Watanabe. Atherosclerosis. 2009;207(1):1–7. 10.1016/j.atherosclerosis.2009.03.024 19389675

[25] Morishige K, Kacher DF, Libby P, Josephson L, Ganz P, Weissleder R, et al High-Resolution Magnetic Resonance Imaging Enhanced With Superparamagnetic Nanoparticles Measures Macrophage Burden in Atherosclerosis. Circulation. 2010;122(17):1707–15. 10.1161/CIRCULATIONAHA.109.891804 20937980 PMC3003265

[26] Korosoglou G, Weiss RG, Kedziorek DA, Walczak P, Gilson WD, Schär M, et al Noninvasive Detection of Macrophage-Rich Atherosclerotic Plaque in Hyperlipidemic Rabbits Using “Positive Contrast” Magnetic Resonance Imaging. Journal of the American College of Cardiology. 2008;52(6):483–91. 10.1016/j.jacc.2008.03.063 18672170 PMC2628468

[27] Deguchi J-o, Aikawa M, Tung CH, Aikawa E, Kim DE, Ntziachristos V, et al Inflammation in Atherosclerosis: Visualizing Matrix Metalloproteinase Action in Macrophages In Vivo. Circulation. 2006;114(1):55–62. 16801460 10.1161/CIRCULATIONAHA.106.619056

[28] Sanz J, Fayad ZA. Imaging of atherosclerotic cardiovascular disease. Nature. 2008;451(7181):953–7. 10.1038/nature06803 18288186

[29] Libby P. Inflammation in atherosclerosis. Nature. 2002;420(6917):868–74. 12490960 10.1038/nature01323

[30] Wu YL, Ye Q, Foley LM, Hitchens TK, Sato K, Williams JB, et al In situ labeling of immune cells with iron oxide particles: An approach to detect organ rejection by cellular MRI. Proceedings of the National Academy of Sciences of the United States of America. 2006;103(6):1852–7. 16443687 10.1073/pnas.0507198103 PMC1413627

[31] Schmitz SA, Coupland SE, Gust R, Winterhalter S, Wagner S, Kresse M, et al Superparamagnetic Iron Oxide–Enhanced MRI of Atherosclerotic Plaques in Watanabe Hereditable Hyperlipidemic Rabbits. Investigative Radiology. 2000;35(8):460–71. 10946973 10.1097/00004424-200008000-00002

[32] Lim E-K, Jang E, Kim J, Lee T, Kim E, Park HS, et al Self-fabricated dextran-coated gold nanoparticles using pyrenyl dextran as a reducible stabilizer and their application as CT imaging agents for atherosclerosis. Journal of Materials Chemistry. 2012;22(34):17518–24.

[33] Ruehm SG, Corot C, Vogt P, Kob S, Debatin JF. Magnetic Resonance Imaging of Atherosclerotic Plaque With Ultrasmall Superparamagnetic Particles of Iron Oxide in Hyperlipidemic Rabbits. Circulation. 2001;103(3):415–22. 11157694 10.1161/01.cir.103.3.415

[34] Sasaki T, Kuzuya M, Nakamura K, Cheng XW, Shibata T, Sato K, et al A Simple Method of Plaque Rupture Induction in Apolipoprotein E–Deficient Mice. Arteriosclerosis, Thrombosis, and Vascular Biology. 2006;26(6):1304–9. 16574894 10.1161/01.ATV.0000219687.71607.f7

[35] Howles GP, Ghaghada KB, Qi Y, Mukundan S Jr, Johnson GA. High-resolution magnetic resonance angiography in the mouse using a nanoparticle blood-pool contrast agent. Magnetic Resonance in Medicine. 2009;62(6):1447–56. 10.1002/mrm.22154 19902507 PMC2787905

[36] Park J, An K, Hwang Y, Park J-G, Noh H-J, Kim J-Y, et al Ultra-large-scale syntheses of monodisperse nanocrystals. Nat Mater. 2004;3(12):891–5. 15568032 10.1038/nmat1251

[37] Kim B, Yang J, Lim EK, Park J, Suh J-S, Park HS, et al Double-ligand modulation for engineering magnetic nanoclusters. Nanoscale Res Lett. 2013;8(1):104 10.1186/1556-276X-8-104 23433032 PMC3614429

[38] Platt N, Suzuki H, Kurihara Y, Kodama T, Gordon S. Role for the class A macrophage scavenger receptor in the phagocytosis of apoptotic thymocytes in vitro. Proceedings of the National Academy of Sciences. 1996;93(22):12456–60. 8901603 10.1073/pnas.93.22.12456 PMC38013

