# Supplementary figures and images for: Compensatory UTE/T2W Imaging of Inflammatory Vascular Wall in Hyperlipidemic Rabbits
Source: PLoS One. 2015 May 15;10(5):e0124572. doi: 10.1371/journal.pone.0124572 (PMC4433322; doi:10.1371/journal.pone.0124572)

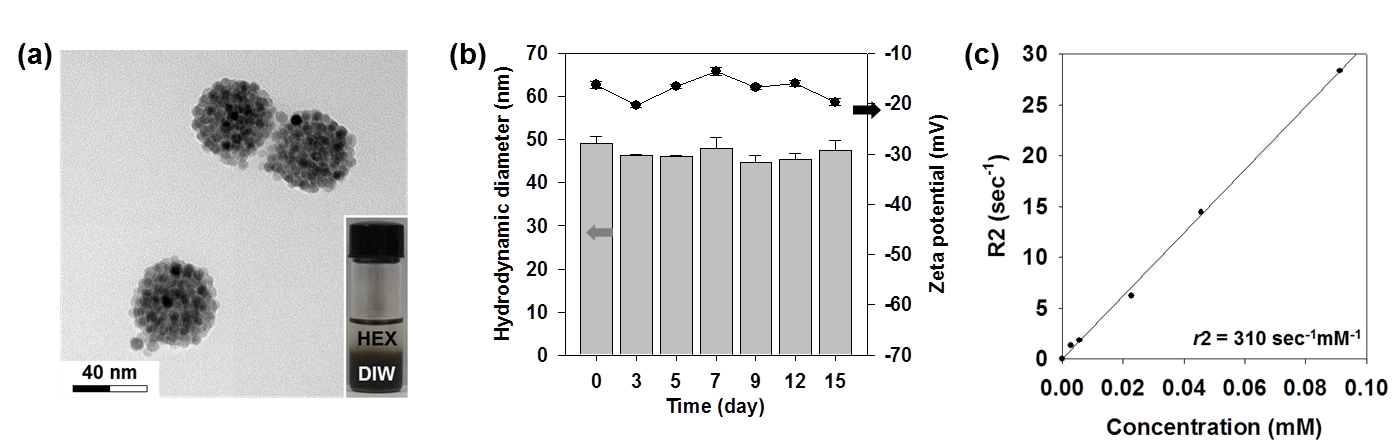

Supplement: S1 Fig — (a) TEM image of DMNC. (b) Size and surface charge variation of DMNC over 15 days. (c) R2 graph of DMNC from solution MR imaging at various Fe concentrations. (TIF) [file pone.0124572.s001.tif]

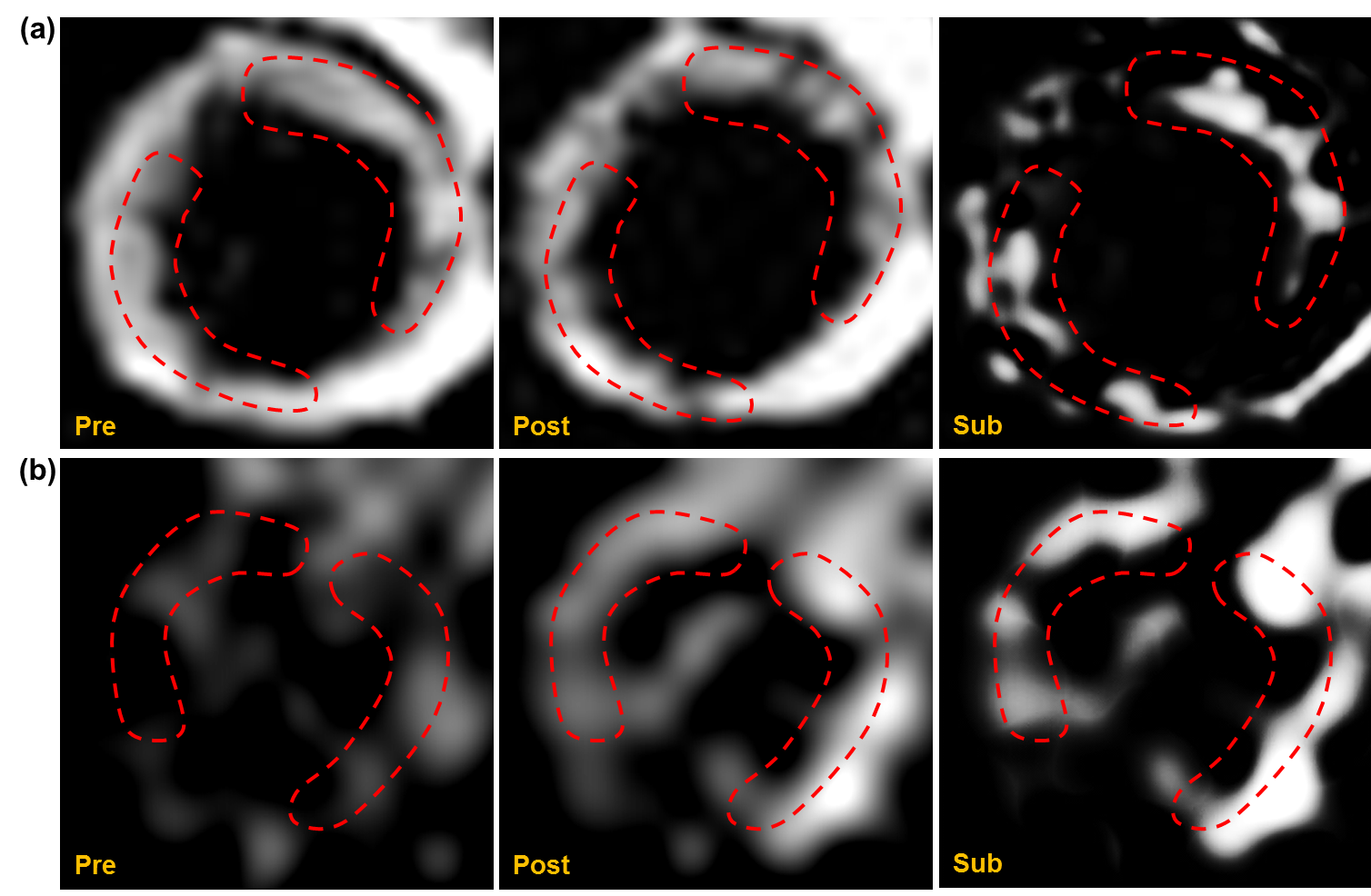

Supplement: S2 Fig — Subtracted images of thoracic aorta using (a) T2W imaging (Sub = Pre—Post) and (b) UTE imaging (Sub = Post—Pre). (TIF) [file pone.0124572.s002.tif]

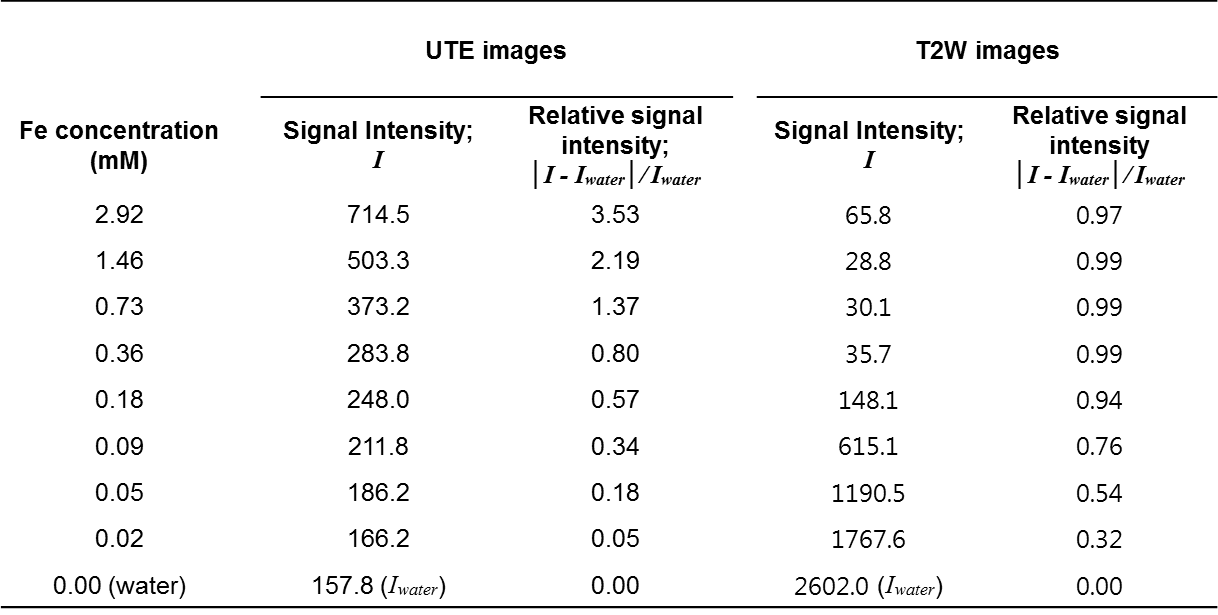

Supplement: S1 Table — (TIF) [file pone.0124572.s004.tif]
