# Supplementary material for: Compensatory UTE/T2W Imaging of Inflammatory Vascular Wall in Hyperlipidemic Rabbits
Source: PLoS One. 2015 May 15;10(5):e0124572. doi: 10.1371/journal.pone.0124572 (PMC4433322; doi:10.1371/journal.pone.0124572)
Supplement: S1 Supporting Text — (DOCX) [file pone.0124572.s003.docx]

S. Supporting file in text.

In S1 Figure, MNP showed monodisperse size (12±0.4 nm) with superparamagnetic property and a saturation magnetization value of of 88 emu g^-1^ Fe at 1.5 T. MNP exhibited water-insolubility with 20.7% weight of fatty acid ligand. DMNC showed uniform and spherical shape and were water-soluble (a). The DMNC was highly stable in the aqueous phase without precipitation for 15 days, with a hydrodynamic diameter of 49.1±1.7 nm and surface charge of -16.3±0.7 mV (b). DMNC showed relaxivity coefficient (*r*2) value of 310 sec^-1^mM^-1^ (c).

In S1 Table, the signal intensity of the DMNC solution in UTE and T2W imaging was measured in each selected ROI. The signal intensity of the DMNC solution in UTE imaging gradually increased up to a Fe concentration of 0.09 mM, and an increase in Fe concentration over 0.09 mM induced a significant signal increase without signal saturation until 2.92 mM. In the T2W images, signal intensity decreased immediately at Fe concentrations of 0.02 mM and greater, but the saturated signal did not decrease further at Fe concentrations greater than 0.18 mM.
